# Editorial: The synergistic effects of nutrition and physical activity on cognitive function and mental health

**DOI:** 10.3389/fnut.2026.2007529

**Published:** 2026-10-27

**Authors:** Shinuk Kim, Tae-Woon Kim, Hyunsik Kang

**Affiliations:** 1 Gyedang College of General Education, Sangmyung University, Cheonan, Republic of Korea; 2 Department of Human Healthcare, Gyeongsang National University, Jinju, Republic of Korea; 3 College of Sport Science, Suwon, Republic of Korea

**Keywords:** diet, lifestyles, mental health, mind-body interventions, nutrition, physical activity

## Introduction

The interaction of lifestyle, nutrition, physical activity, and mental health represents one of the most dynamic and critical areas of modern public health and clinical research. Understanding how lifestyle-related behaviors influence the brain across the human lifespan is paramount as populations face shifting demographic patterns, rising rates of metabolic disorders, and an increasing burden of mental health conditions. A broad spectrum of authors representing physical education, exercise science, nutrition, psychology, and neuroscience have contributed to this Research Topic, introducing lifestyle interventions, mediating pathways, and mental health outcomes. In this editorial, we provide a thematic overview of the interesting and diverse contents of this Research Topic, summarized in Table 1.

**Table 1:** A summary table of the outcomes of 14 papers published in Research Topic “*The synergistic effects of nutrition and physical activity on cognitive function and mental health*.”

Lifestyle interventions	Mediating pathways	Health outcomes
•Nutrition (dietary diversity, fruits/vegetables, ultra-processed) •Physical activity (fitness, dance, body-mind exercises) •Clinical interventions	•Psychological (resilience, loneliness, subjective experiences) •Social (social participation) •Physical/body (BMI, handgrip strength, Body Shape Index)	•Mental health (depression, anxiety, mood disorders) •Cognitive function (executive function, dementia risk) •General wellbeing and sleep
**Nutrition factors** •Ultra-processed foods and dietary diversity (Ren et al., Shen et al.) •Fruit and vegetable intake (Saintila). •Appetite and clinical treatments (Mechlińska et al.).	**Psychological and cognitive mediators** •Psychological resilience and loneliness buffers the path from activity to addictive behaviors like mobile phone use (Ding and Liu) •Subjective exercise experiences links physical activity to lower depression in emerging adults (Shen et al.). •Negative emotions link to specific eating behaviors, moderated by activity (Yue et al.)	**Mental health and mood** •Reduction of anxiety and depressive symptoms (Saintila, Ren et al., Li et al., Yue et al.). •Management of treatment-resistant mood disorders and late-life depression (Mechlińska et al.). •Stabilization of metabolic syndrome risk factors in Schizophrenia (Wang et al.)
**Physical activity and exercise** •Cardiorespiratory fitness (Bi et al.) •Dance interventions and square dancing (Yue et al., Wang et al., Song et al., Li et al.) •Mid-body exercises and general activity (Zhou et al., Shen et al.)	**Social and physical mediators** •Social participation mediates square dancing and older adults wellbeing (Song et al.) •Body composition metrics (BMI, body shape index, low handgrip strength) act as physiological risk (Bi et al.) or mediating markers for metabolic syndrome and dementia (Ramírez-Vélez et al., Shen et al.)	**Cognitive function** •Preservation of executive function and prevention of cognitive impairment or dementia (Bi et al., Ramírez-Vélez et al., Shen et al.)
**Combined physical-nutritional status** • Obesity, BMI, and handgrip strength (Ramírez-Vélez et al., Shen et al.)		**Behavioral and sleep** •Mitigation of mobile phone addiction (Ding and Liu) •Improvement in sleep quality via physical/dance interventions (Li et al.)

## Lifestyle, diet, and mental health

Poor dietary habits and nutritional deficiencies directly contribute to emotional distress and cognitive decline (Saintila). Several studies have demonstrated that the consumption of highly processed foods in conjunction with a sedentary lifestyle is associated with an increase in the severity of depressive symptoms in young adults (Ren et al.). On the other hand, eating a sufficient amount of fruits and vegetables and engaging in physical activity to protect yourself from anxiety and depression (Saintila). The preservation of cognitive function in older adults can be achieved through the maintenance of a high level of dietary diversity and consistent physical activity over the course of time. In the meantime, targeted nutritional interventions offer promising clinical tools for the management of depression in later-life-stage patients (Li et al.).

## Physical activity, exercise, and addictions

In older age, negative emotions have a direct influence on eating behaviors, and physical activity plays a vital role as a mediator in this pathway (Yue et al.). The administration of ketamine opens up new perspectives on appetite changes that occur during treatment-resistant mood disorders (Mechlińska et al.). Emerging adults also experience shifts in mood that undergo mood shifts associated with physical activity and their physical activity and subjective exercise experiences (Shen et al.). The combination of creative interventions and physical activity is important for promoting mental health (Ding and Liu). The ability to move around and engage in physical activity is a powerful protective factor against psychological distress and addiction. Teenagers who engage in physical activity have a lower likelihood of becoming addicted to mobile phones, with psychological resilience and loneliness serving as key modulating factors (Ding and Liu). Another factor that influences executive brain function in young people is the combination of body shape and cardiorespiratory fitness (Bi et al.).

## Dance and mind-body interventions

Mind-body movements like dance, yoga, and tai chi serve as highly effective non-pharmacological treatments. They simultaneously target the biological (metabolic health, sleep quality), psychological (anxiety, depression), and social (community connection, happiness) aspects of recovery (Wang et al.). Research has demonstrated that square dancing enhances social wellbeing and fosters cultural connections among older adults (Song et al.). Additionally, general dance interventions have been shown to improve the overall quality of sleep (Li et al.). These interventions offer accessible, low-risk, and highly scalable solutions for diverse populations—ranging from severe psychiatric patients (schizophrenia) and chronic disease survivors (breast cancer) to aging populations seeking community (Zhou et al.). Moreover, combining physical weakness (low handgrip strength) and obesity significantly increases the risk of cognitive impairment and dementia (Ramírez-Vélez et al., Shen et al.).

## Conclusions

The collective implication of these studies is clear: lifestyle interventions should no longer be treated as secondary, “complementary” advice. Nutrition, structured movement, social physical activities, and muscle preservation are foundational, front-line medicine. To combat the rising global crises of mental illness, addiction, and dementia, healthcare systems must aggressively integrate lifestyle medicine into standard clinical practice.

